# Vagus Nerve Stimulation in Stroke Management: Brief Review of Evolution and Present Applications Paired with Rehabilitation

**DOI:** 10.3390/brainsci15040346

**Published:** 2025-03-27

**Authors:** Prasad S. Vannemreddy, Mark Cummings, Romana V. Bahrii, Konstantin V. Slavin

**Affiliations:** 1 Department of Neurosurgery, University of Illinois at Chicago, Chicago, IL 60612, USA; 2 Brain Plasticity Laboratory, Department of Physical Therapy, College of Applied Health Sciences, University of Illinois at Chicago, Chicago, IL 60612, USA; mcummi8@uic.edu; 3 Graduate Program in Rehabilitation Sciences, College of Applied Health Sciences, University of Illinois at Chicago, Chicago, IL 60612, USA; 4 Neurology Section, Jesse Brown Veterans Administration Medical Center, Chicago, IL 60612, USA

**Keywords:** stroke, paralysis, vagus nerve, VNS, physical therapy

## Abstract

Cerebrovascular accident (CVA) or stroke is a devastating neurological condition with dismal prognosis associated with recurrent episodes that further damage the neuronal networks, thus disabling neuronal plasticity. Vagus nerve stimulation (VNS) has been used in clinical practice to treat epilepsy for several decades and is well accepted as a safe procedure devoid of serious adverse events. Bailey and Bremer demonstrated that VNS has the capabilities to stimulate neuronal pathways that enhance the recovery of damaged cerebral function. Further studies have strengthened these observations, while technology has improved the tolerability of implants, resulting in VNS applications for epilepsy. Several animal models on neural plasticity have improved our understanding of VNS and its ability to provide neuromodulation to improve recovery in stroke patients. The closed-loop stimulation of the vagus nerve with individualized stimulation parameters combined with physical therapy appears to be an attractive option today. VNS is also being tested as a noninvasive trans-cutaneous modality to further improve patient acceptance and tolerability. However, the implantation of VNS is yielding desirable outcomes and appears to be a more reliable treatment for stroke rehabilitation in clinical trials.

## 1. Introduction

**Figure 1:**
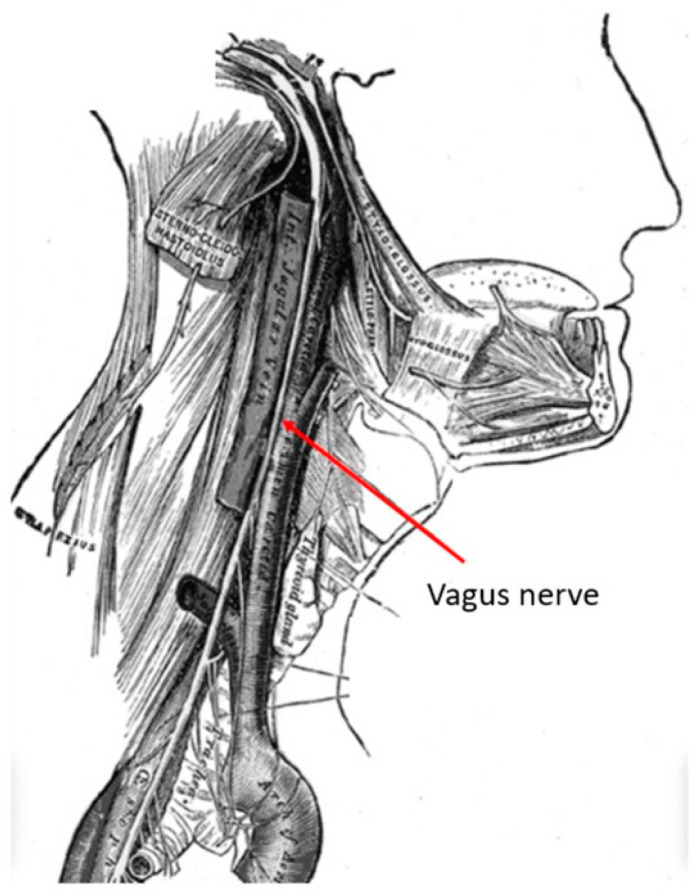
Anatomy of the vagus nerve in the neck within the carotid sheath. This anatomy if very familiar to neurosurgeons who perform anterior cervical spine surgery. (Courtesy: creative commons).

The vagus nerve is the longest cranial nerve, providing bilateral parasympathetic innervation to the viscera. It is located in the posterior layer of the carotid sheath within the cervical deep fascia of the neck (Figure 1). Its extensive clinical utility became evident following electrical stimulation studies by Bailey and Bremer in 1938 in Chicago, IL, USA [1]. Their observations were later reproduced by Dell and Olson, whose feline studies on vagal nerve projections to the sensory cortex, thalamus and cerebellum demonstrated the potential of vagus nerve stimulation (VNS) in synchronizing cortical activity [2]. A detailed examination with recent technology has named these network connections as the “vagus afferent network”, part of connectomics. These extensive vagus nerve afferents include neurotransmitter centers like the nucleus tractus solitarius, locus coeruleus, dorsal raphe nucleus, parabrachial nucleus, thalamocortical connections, limbic system circuits of amygdala, hippocampus, hypothalamus and cerebellum [3].

## 2. Vagus Nerve as a Neuromodulation Target

### 2.1. Anatomy of Vagus Nerve and Neuromodulatory Projections

The vagus nerve provides the primary parasympathetic supply to the body, encompassing both sensory and motor innervation. In the neck, it primarily consists of ascending fibers that enable access to the cortical centers via electrical stimulation through the nuclei of the solitary tract, the locus coeruleus, raphe nuclei, and nucleus basalis—key sources for neuromodulatory chemicals like acetyl choline, norepinephrine, and serotonin [4,5,6,7].

These nuclei have extensive connections throughout the central nervous system, with preferential supply to certain brain regions. The locus coeruleus (LC) supplies adrenergic fibers to the prefrontal, motor and sensory cortices [8], while the raphe nuclei (RN) provide serotonergic innervation to the visual cortex and other primary sensory cortices [9].

Additionally, the limbic system receives cholinergic input from the nucleus basalis (NB) [10], whereas spinal cord interneurons receive serotonergic and adrenergic input from the LC and RN [11]. Consequently, given these widespread connections, the vagus nerve has the unique ability to control or modulate these extensive areas of the nervous system [12,13].

### 2.2. Neural Mechanisms of Paired VNS

Neuromodulation by VNS combined with physical rehabilitation is better known as paired VNS. The aforementioned neurotransmitters affect both the acute excitability and neuroplasticity of the CNS [14,15]. Inhibiting the release of these neurotransmitters prevented paired VNS-induced cortical reorganization in animal models [16,17], resulting in behavioral alterations [18]. Multiple studies have illustrated the potential of paired VNS-induced neuromodulation to enhance neuronal plasticity and regeneration in clinical populations including chronic stroke [19]. Many neurotransmitters, especially dopamine, encourage learning patterns to remodel behavior in a positive direction. This reinforcement is considered a key mechanism for initiating neurorehabilitation [20,21]. Evidence from animal studies suggests that VNS delivered after training provides targeted reinforcement, whereas stimulation alone results in the diffuse activation of neuronal output. In rats, Engineer et al. (2011) demonstrated that combining vagus nerve stimulation with training yielded increased responses in the auditory cortex [22], similar to findings from studies involving the motor activity of the forelimbs [17,23]. However, timing is crucial when combining stimulations to match synaptic activity at the neuronal level for optimal results in stroke rehabilitation [24].

Several studies emphasized the importance of precise timing in paired VNS since delays in reinforcement can dampen its effectiveness [16,23]. VNS may also play a role in reducing inflammatory responses and blood–brain barrier (BBB) permeability; however, in a chronic stroke patient set for rehabilitation, these acute mechanisms may not be relevant [25].

### 2.3. Vagus Nerve and Clinical Neuromodulation

Indications for VNS in clinical settings have existed in the field of epilepsy and later in refractory cases of depression, while more recently, its potential has expanded to stroke rehabilitation, obesity treatment and various neuropsychiatric diseases [26,27].

Future indications for VNS may include pain, migraine, cardiovascular disease, and inflammatory/autoimmune diseases [28,29,30]. These novel applications for VNS are being actively investigated, driven by advancements in less invasive devices, improved surgical techniques of implantation [31], and recently developed closed-loop systems [32].

### 2.4. The VNS Equipment

**Figure 2:**
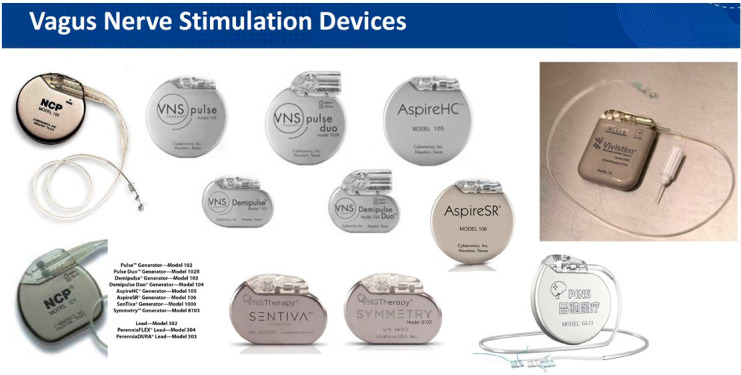
Various commercially available VNS equipment versions. Note the recognizable difference in the appearance of VNS devices used for epilepsy and depression and the dedicated device (last on the right) for post-stroke rehabilitation.

Zabara et al. have been credited with introducing the modern VNS system, which consists of a pulse generator connected by a wire to the electrode wrapped around the vagus nerve within the carotid sheath in the neck [33]. After implantation, the system is typically activated after surgical trauma subsides, 2 weeks postoperatively, and then the stimulation parameters are adjusted to optimize clinical outcomes and patient comfort [34]. While the electrode design has remained unchanged over the years, the generator (Figure 2) has undergone several updates, becoming more sophisticated and miniaturized. These advancements have reduced surgical trauma and hospitalization while improving patient acceptance without compromising treatment efficacy. Noninvasive VNS alternatives include the transcutaneous stimulation of the main trunk of the vagus nerve in the neck (tcVNS) or the auricular branch of the vagus nerve (ABVN) by keeping electrodes on the earlobe (taVNS).

### 2.5. Mechanisms of VNS

The vagus nerve is a major component of the parasympathetic innervation of the viscera, predominantly consisting of afferent fibers. Hence, its stimulation has yielded various therapeutic effects across a variety of disorders [28,35]. Zabara demonstrated the antiepileptic influence of VNS in canines, while Krahl et al. proposed that the inhibition or lesioning of the LC may be responsible for the suppression of seizure activity [33,36]. It has also been postulated that the noninvasive percutaneous technique mimics the standard implanted version of VNS.

### 2.6. Neural Substrate

The locus coeruleus, the brain’s primary noradrenergic center, receives projections from the vagus nerve, and its ablation reduces seizure activity, similar to VNS [36]. In addition, VNS modulates monoaminergic activity by increasing both serotonin and norepinephrine concentrations in CSF [37]. Moreover, in rats, VNS enhances the electrical activity of monoaminergic neurons, a major event in depression management [38]. Neuroimaging studies indicate that VNS influences the prefrontal cortex and limbic system, suggesting a role in mood regulation. Additionally, VNS has been shown to increase brain-derived neurotrophic factor (BDNF) expression, which plays a crucial role in alleviating depression and facilitating post-stroke motor activity as an essential component in enhancing learning and memory [39,40,41]. Driskill et al. studied drug-seeking rat models to demonstrate that VNS increased BDNF in the infralimbic cortex to regulate synaptic activity for neuronal plasticity [42].

## 3. Clinical Applications

### 3.1. Neuromodulation in Stroke Rehabilitation

Cerebrovascular accidents or strokes remain a major burden on health care, ranking among the leading causes of mortality and morbidity [43]. Surviving stroke patients struggle to regain the functional use of their paralyzed body parts, and motor rehabilitation serves a critical role in improving their quality of life. Zhi et al. (2022) found neuromodulation most efficient among four motor rehabilitation treatments (neuromodulation, training, technological and pharmacological intervention) to improve function post-stroke [44]. Despite their initial promise, current neuromodulatory options have been insufficient to improve motor function following a stroke. Following the initial study by Bailey and Bremer [1], the stimulation of the vagus nerve has been investigated in several animal models of stroke to evaluate recovery patterns. Utilizing motor as well as non-motor tasks, experimental results have been promising, demonstrating that pairing VNS with the concerned activity (motor or auditory tasks) enhances cortical reorganization and improves the plasticity in the relevant cortical areas [45,46]. Additionally, VNS modulates immune responses and has shown to improve outcomes not only in stroke recovery but also in post-traumatic stress disorder (PTSD) therapy [47,48].

### 3.2. Implanted VNS and Paired Motor Rehabilitation Therapy

Patients in trials receiving physical rehabilitation with or without VNS (uniform stimulation parameters of, 0.8 mA current amplitude, 0.1 ms pulse width, 30 Hz frequency, and 0.5 s duration, delivered along with simultaneous motor movements) exhibited meaningful arm recovery favoring stimulation in several trials [27,49,50]. As recently as 2021, the FDA approved the Vivistim Paired VNS system by MicroTransponder (Vivistim, MicroTransponder Inc., Austin, TX, USA) for the treatment of moderate to severe upper extremity motor deficits associated with chronic ischemic stroke. Several ongoing multicenter studies are being conducted to continue investigating its efficacy.

### 3.3. Technique of VNS Implantation

**Figure 3:**
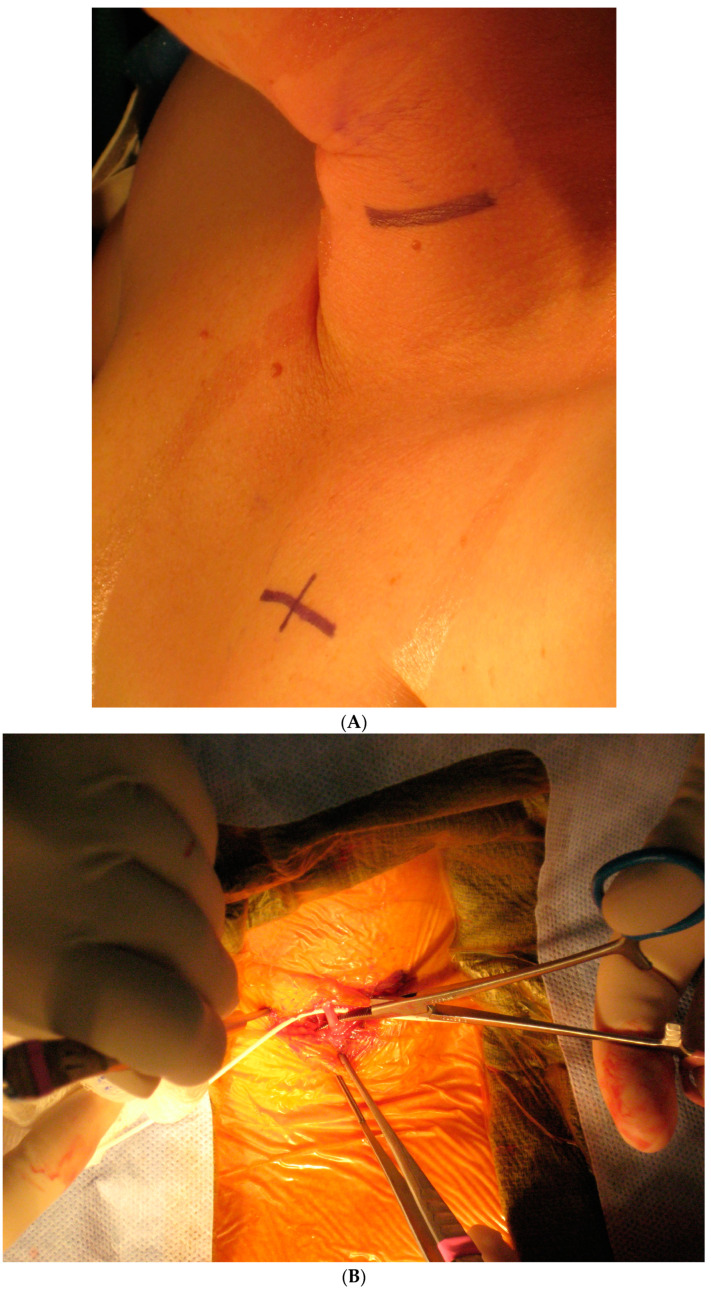

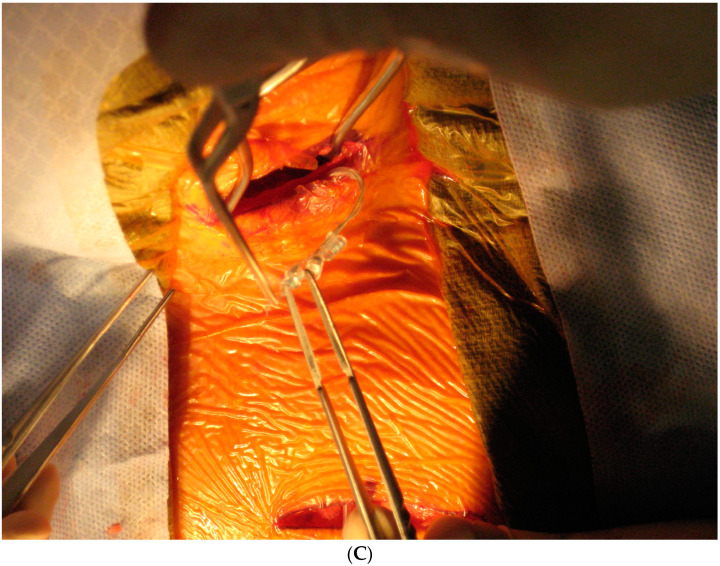
Implantation steps of the VNS system: (**A**) the marking of skin incisions in the neck and infraclavicular region; (**B**) the dissection of the vagus nerve in the cervical region; (**C**) the handling of the VNS electrode prior to the implantation.

Today, VNS implantation is a standardized procedure following the routine approach for epilepsy surgery. It is performed on the left side only. After opening the skin and platysma, a carotid triangle is dissected to reach the posterior layer of the carotid sheath where the vagus nerve is located. The nerve is freed along its longitudinal extent to wrap the spring electrode around it (Figure 3). This electrode has extensions for the implantable pulse generator (IPG). A separate incision is given in the infraclavicular region to accommodate the IPG and a subcutaneous tunnel is made to connect both the incisions and bring down the electrode extensions. The battery is placed in the infraclavicular region and connected to these electrodes. Incisions are closed in layers after ensuring a favorable signal from the remote operator.

**Figure 4:**
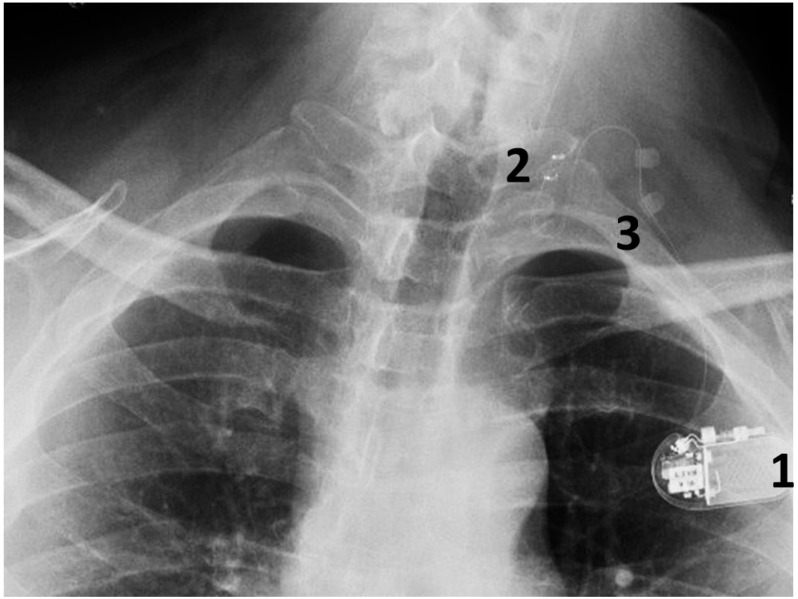
A chest radiograph showing the components of the implanted VNS system: 1. The implantable pulse generator; 2. the electrode contacts on the left vagus nerve; 3. silicone anchors to stabilize the electrode in place.

This is conducted under general anesthesia, and the patient is kept under observation for a day. The surgical procedure is safe and serious complications are rare (Figure 4).

In a long-term follow-up review of 497 procedures, the team of Ben-Menachem reported an overall complication rate of 8.6% for all implantation procedures and 3.7% were hardware-related complications. Postoperative hematoma and infection were the most common complications; others included infection, vocal cord palsy, facial weakness, pain and the disconnection of the leads [51].

### 3.4. Noninvasive VNS

Noninvasive VNS, specifically taVNS, has been explored in stroke rehabilitation using slightly differing stimulation parameters [52,53,54]. Targeting cymba concha or acoustic meatus on the left side, one of these studies yielded significant improvements in upper extremity motor function [53]. Redgrave et al. utilized 18 sessions of 1 h each applying 25 Hz stimulation and 100 s pulse width for taVNS in post-stroke patients [53]. Baig et al. employing a similar technique in chronic stroke patients obtained promising recovery in sensory loss too [55]. Other trials also had encouraging outcomes favoring stimulation over sham stimulation [52,54]. Capone et al. combined robotic-assisted physical therapy with taVNS for rehabilitation of their post-stroke patients to document that noninvasive VNS resulted in significantly greater motor recovery compared to physical rehabilitation alone [52].

In a pilot study, motor-activated auricular vagus nerve stimulation (MAAVNS) was encouraging in its outcomes. In an individualized closed-looped stimulation study, MAAVNS was used to improve upper extremity motor function, utilizing surface electromyography (EMG) for the stimulation delivery feedback [33]; in this pilot study on 16 patients, Badran et al. came up with encouraging results showing noninvasive tcVNS treatment to be comparable to the implanted VNS [33].

Peng et al. utilized neuroimaging (fMRI) to evaluate ipsilateral versus contralateral ear stimulation by measuring blood oxygenation level-dependent (BOLD) signals [56]. The study demonstrated that the same side ear stimulation (ipsilateral neuromodulation) provided a greater excitation of the ipsilateral cortex and stronger activation of task-related motor areas compared to contralateral neuromodulation.

## 4. Discussion

Current estimates reveal that 7.6 million adults are struggling with stroke in the US, while an additional 3.4 million will join this group in ten years [57]. The majority of these patients suffer from sustained upper extremity motor dysfunction, curtailing their activities of daily living (ADLs) and quality of life [58,59,60]. Over the past decade, physical therapy and rehabilitation techniques have evolved with some promise but remain time-intensive and require specialized environments with certain equipment [61,62,63]. However, neuromodulation by VNS combined with physical rehabilitation (paired VNS) appears to be a more efficient treatment option [64].

Chicago neurosurgeon Percival Bailey conducted extensive laboratory studies on cerebral localization and functional neurosurgery. Animal experiments by Bailey and Bremer revealed that the stimulation of the vagus nerve enhanced memory via afferent projections to the brainstem [1]. Subsequent experimental and preclinical studies further suggested the role of VNS in promoting neuronal plasticity following neurological injury [19,65]. Study groups in stroke models demonstrated consistent improvements compared to control groups when VNS was applied simultaneously with physical therapy [65].

Translational studies have established possible pathways through which paired VNS activity improves the paralyzed upper extremity in humans: the descending ipsilateral corticospinal tract and the contralateral cortico-reticulospinal tract [66]. Meyers et al. demonstrated that paired VNS significantly improved upper limb function in paretic rats compared to sham stimulation [18,67]. It is also possible that other motor pathway regions may contribute to improving neural plasticity following paired VNS.

### 4.1. VNS and the Stimulation Parameters in Human Stroke Rehabiliation

The vagus nerve is stimulated on the left side for VNS and the parameters need to be tailored to patient comfort rather than the elicitation of the desirable response. The increasing intensity may improve the stimulation of the nerve, but the patient’s tolerance should take the priority as discomfort can cause disproportionate impedance. Here, the pulse width requires attention along with its interaction with stimulation intensity. Increasing the pulse width can achieve better VNS results, keeping the intensity stable [68]. There is still scope for exploring the interactions between stimulation and pulse width.

Ben-Menachem et al. recommended a biologically active frequency between 20 and 30 Hz for both implantation and transcutaneous VNS for optimum behavior results, pending a further search for acceptable frequency levels [69]. Other investigators also concede the need to study the ranges of frequencies as well as the on/off periods to prevent damage to the nerve and prolong the IPG duration of life without compromising the neural plasticity effects of the therapy [70].

### 4.2. The Noninvasive Transcutaneous Stimulation of the Vagus Nerve

Transcutaneous stimulation methods differ significantly from the implanted VNS systems in both their neuromodulation pathways and stimulation responses [71].

The noninvasive methodology predominantly elicits responses mediated by a cutaneous sensation and may not even reach the vagus nerve fibers within the carotid sheath. Understandably, the percutaneous stimulation tends to fail in eliciting physiological activity comparable to standard VNS. Bucksot et al. demonstrated that tcVNS may achieve responses similar to implanted VNS, but only at much higher stimulation intensities—potentially exceeding clinical tolerability to a great extent [72]. Additionally, tcVNS may not provide adequate stimulation consistency to yield the desired clinical results. Thus, stimulation intensity must be modulated to moderate intensities in order to be clinically efficient and tolerable [19]. One possible reason for this failure is the anatomical variability of the vagus nerve at stimulation sites, which may contribute to inconsistent effects [73,74].

### 4.3. Safety and Feasibility of VNS

VNS has been in clinical use for several decades and has proven to be safe and well tolerated, especially in epilepsy patients. In stroke patients, so far, it has been reported to be safe without serious adverse events related to the procedure or the implant [75]. Its utility as a combination therapy in stroke rehabilitation is another encouraging feature since it may reduce the number of required visits and allow for personalized device monitoring. Multicenter clinical trials have been approved by the FDA to further evaluate the efficacy of paired VNS in motor rehabilitation for stroke patients.

### 4.4. Other Promising Indications for Paired VNS

Beyond the motor improvements observed in stroke patients, paired VNS shows potential for broader neuromodulation applications, including incomplete cervical spinal cord injury, lower extremity paralysis, dysphagia and sphincter dysfunctions [76,77].

## 5. Summary

Paired VNS therapy used in stroke rehabilitation is a safe and promising modality, with encouraging outcomes from multiple multi-institutional studies supporting its clinical use. Combining rehabilitation-aimed motor activity and neuromodulation appears to be a better approach for improving motor function than the passive physical therapy of a paralyzed extremity alone [63] while also reducing compensatory movements [78]. Based on robust supporting preclinical models, VNS for stroke rehabilitation is tailored to the requirements of the paralyzed extremity, the upper extremity as of now, especially when delivered through an individualized closed loop of short bursts of stimulation synchronized with physical therapy movements [79]. This therapy is supported by the observations that paired VNS activates several neuromodulation pathways engaging noradrenergic, cholinergic and serotonergic networks to improve neuronal plasticity [19]. While noninvasive VNS might be appealing to the patients, its mechanism may differ from that of implanted neuromodulatory systems. At this point in time, there is no consensus on the possible best parameter for VNS therapy, considering the multiple variables at play during the rehabilitation of the stroke patients. Hopefully, a wide range of stimulation frequencies, pulse widths, current intensities and durations will be narrowed down to contribute to the long-lasting outcomes in VNS treatment in human neurological ailments.

## Data Availability

Not applicable.

## Abbreviations

The following abbreviations are used in this manuscript:

CVA	Cerebrovascular accident
VNS	Vagal nerve stimulation
CNS	Central nervous system
LC	Locus coeruleus
RN	Raphe nuclei
tcVNS	Transcutaneous VNS
ABVN	Auricular branch of the vagus nerve
taVNS	Transauricular VNS
CSF	Cerebrospinal fluid
BDNF	Brain-derived neurotrophic factor
PTSD	Post-traumatic stress disorder
MAAVNS	Motor-activated auricular VNS
fMRI	Functional magnetic resonance imaging
BOLD	Blood oxygenation level-dependent
ADL	Activity of daily living

## References

[1] Bailey P., Bremer F. (1938). A sensory cortical representation of the vagus nerve with a note on the effects of low blood pressure on the cortical electrogram. J. Neurophysiol..

[2] Dell P., Olson R. (1951). Thalamic, cortical and cerebellar projections of vagal visceral afferences. C. R. Seances Soc. Biol. Fil..

[3] Hachem L.D., Wong S.M., Ibrahim G.M. (2018). The vagus afferent network: Emerging role in translational connectomics. Neurosurg. Focus.

[4] Beckstead R.M., Norgren R. (1979). An autoradiographic examination of the central distribution of the trigeminal, facial, glossopharyngeal, and vagal nerves in the monkey. J. Comp. Neurol..

[5] Zhukova G.P. (1980). The afferent pathway to the locus coeruleus from the nucleus of the solitary tract. Neurosci. Behav. Physiol..

[6] Herbert H. (1992). Evidence for projections from medullary nuclei onto serotonergic and dopaminergic neurons in the midbrain dorsal raphe nucleus of the rat. Cell Tissue Res..

[7] Semba K., Reiner P.B., Mcgeer E.G., Fibiger H.C. (1988). Brainstem afferents to the magnocellular basal forebrain studied by axonal transport, immunohistochemistry, and electrophysiology in the rat. J. Comp. Neurol..

[8] Chandler D.J., Gao W.J., Waterhouse B.D. (2014). Heterogeneous organization of the locus coeruleus projections to prefrontal and motor cortices. Proc. Natl. Acad. Sci. USA.

[9] Wilson M.A., Molliver M.E. (1991). The organization of serotonergic projections to cerebral cortex in primates: Regional distribution of axon terminals. Neuroscience.

[10] Mesulam M.M., Geula C. (1988). Nucleus basalis (Ch4) and cortical cholinergic innervation in the human brain: Observations based on the distribution of acetylcholinesterase and choline acetyltransferase. J. Comp. Neurol..

[11] Westlund K.N., Dan Coulter J. (1980). Descending projections of the locus coeruleus and subcoeruleus/medial parabrachial nuclei in monkey: Axonal transport studies and dopamine-beta-hydroxylase immunocytochemistry. Brain Res..

[12] Dorr A.E., Debonnel G. (2006). Effect of vagus nerve stimulation on serotonergic and noradrenergic transmission. J. Pharmacol. Exp. Ther..

[13] Hulsey D.R., Riley J.R., Loerwald K.W., Rennaker R.L., Kilgard M.P., Hays S.A. (2017). Parametric characterization of neural activity in the locus coeruleus in response to vagus nerve stimulation. Exp. Neurol..

[14] Elliott P., Wallis D.I. (1992). Serotonin and L-norepinephrine as mediators of altered excitability in neonatal rat motoneurons studied in vitro. Neuroscience.

[15] Gu Q. (2002). Neuromodulatory transmitter systems in the cortex and their role in cortical plasticity. Neuroscience.

[16] Bowles S., Hickman J., Peng X., Williamson W.R., Huang R., Washington K., Donegan D., Welle C.G. (2022). Vagus nerve stimulation drives selective circuit modulation through cholinergic reinforcement. Neuron.

[17] Hulsey D.R., Shedd C.M., Sarker S.F., Kilgard M.P., Hays S.A. (2019). Norepinephrine and serotonin are required for vagus nerve stimulation directed cortical plasticity. Exp. Neurol..

[18] Meyers E.C., Kasliwal N., Solorzano B.R., Lai E., Bendale G., Berry A., Ganzer P.D., Romero-Ortega M., Rennaker R.L., Kilgard M.P. (2019). Enhancing plasticity in central networks improves motor and sensory recovery after nerve damage. Nature Commun..

[19] Hays S.A., Rennaker R.L., Kilgard M.P. (2023). How to fail with paired VNS therapy. Brain Stimul..

[20] Schultz W. (2015). Neuronal reward and decision signals: From theories to data. Physiol. Rev..

[21] He K., Huertas M., Hong S.U.Z., Tie X., Hell J.W., Shouval H., Kirkwood A. (2015). Distinct eligibility traces for LTP and LTD in cortical synapses. Neuron.

[22] Engineer N.D., Riley J.R., Seale J.D., Vrana W.A., Shetake J.A., Sudanagunta S.P., Borland M.S., Kilgard M.P. (2011). Reversing pathological neural activity using targeted plasticity. Nature.

[23] Khodaparast N., Hays S.A., Sloan A.M., Fayyaz T., Hulsey D.R., Rennaker R.L., Kilgard M.P. (2014). Vagus nerve stimulation delivered during motor rehabilitation improves recovery in a rat model of stroke. Neurorehabil. Neural Repair.

[24] Yagishita S., Hayashi-Takagi A., Ellis-Davies G.C.R., Urakubo H., Ishii S., Kasai H. (2014). A critical time window for dopamine actions on the structural plasticity of dendritic spines. Science.

[25] Andalib S., Divani A.A., Ayata C., Baig S., Arsava E.M., Topcuoglu M.A., Cáceres E.L., Parikh V., Desai M.J., Majid A. (2023). Vagus nerve stimulation in ischemic stroke. Curr. Neurol. Neurosci. Rep..

[26] Austelle C.W., O’Leary G.H., Thompson S., Gruber E., Kahn A., Manett A.J., Short B., Badran B.W. (2021). A comprehensive review of vagus nerve stimulation for depression. Neuromodulation.

[27] Dawson J., Pierce D., Dixit A., Kimberley T.J., Robertson M., Tarver B., Hilmi O., McLean J., Forbes K., Kilgard M.P. (2016). Safety, feasibility, and efficacy of vagus nerve stimulation paired with upper-limb rehabilitation after ischemic stroke. Stroke.

[28] Koopman F.A., Chavan S.S., Milijko S., Tak P.P. (2016). Vagus nerve stimulation inhibits cytokine production and attenuates disease severity in rheumatoid arthritis. Proc. Natl. Acad. Sci. USA.

[29] Shao P., Li H., Jiang J., Guan Y., Chen X., Wang Y. (2023). Role of vagus nerve stimulation in the treatment of chronic pain. Neuroimmunomodulation.

[30] Zhang Y., Popovic Z.B., Bibevski S., Fakhry I., Sica D.A., Van Wagoner D.R., Mazgalev T.N. (2009). Chronic vagus nerve stimulation improves autonomic control and attenuates systemic inflammation and heart failure progression in a canine high-rate pacing model. Circ. Heart Fail..

[31] Badran B.W., Dowdle L.T., Mithoefer O.J., LaBate N.T., Coatsworth J., Brown J.C., DeVries W.H., Austelle C.W., McTeague L.M., George M.S. (2018). Neurophysiologic effects of transcutaneous auricular vagus nerve stimulation (taVNS) via electrical stimulation of the tragus: A concurrent taVNS/fMRI study and review. Brain Stimul..

[32] Badran B.W., Peng X., Baker-Vogel B., Hutchison S., Finetto P., Rishe K., Fortune A., Kitchens E., O’Leary G.H., Short A. (2023). Motor activated auricular vagus nerve stimulation as a potential neuromodulation approach for poststroke motor rehabilitation: A pilot study. Neurorehabil. Neural Repair.

[33] Zabara J. (1992). Inhibition of experimental seizures in canines by repetitive vagal stimulation. Epilepsia.

[34] Penry J.K., Dean J.C. (1990). Prevention of intractable partial seizures by intermittent vagal stimulation in humans: Preliminary results. Epilepsia.

[35] George M.S., Nahas Z., Bohning D.E., Lomarev M., Denslow S., Osenbach R., Ballenger J.C. (2000). Vagus nerve stimulation: A new form of therapeutic brain stimulation. CNS Spectr..

[36] Krahl S.E., Clark K.B., Smith D.C., Browning R.A. (1998). Locus coeruleus lesions suppress the seizure-attenuating effects of vagus nerve stimulation. Epilepsia.

[37] Ben-Menachem E., Hamburger A., Hedner T., Hammond E.J., Uthman B.M., Slater J., Treig T., Stefan H., Ramsay R.E., Wernicke J.F. (1995). Effects of vagus nerve stimulation on amino acids and other metabolites in the CSF of patients with partial seizures. Epilepsy Res..

[38] Follesa P., Biggio F., Gorini G., Caria S., Talani G., Dazzi L., Puligheddu M., Marrosu F., Biggio G. (2007). Vagus nerve stimulation increases norepinephrine concentration and the gene expression of BDNF and bFGF in the rat brain. Brain Res..

[39] Furmaga H., Carreno F.R., Frazer A. (2012). Vagal nerve stimulation rapidly activates brain-derived neurotrophic factor receptor TrkB in rat brain. PLoS ONE.

[40] Khodaparast N., Hays S.A., Sloan A.M., Hulsey D.R., Ruiz A., Pantoja M., Rennakar R.L., Hays S.A. (2013). Vagus nerve stimulation during rehabilitative training improves forelimb strength following ischemic stroke. Neurobiol. Dis..

[41] Liu W., Wang X., O’Connor M., Wang G., Han F. (2020). Brain-derived neurotrophic factor and its potential therapeutic role in stroke comorbidities. Neural Plast..

[42] Driskill C.M., Childs J.E., Phensy A., Rodriguez S.R., O’Brien J.T., Lindquist K.L., Naderi A., Bordleanu B., McGinty J., Kroener S. (2024). Vagus Nerve Stimulation (VNS) modulates synaptic plasticity in the infralimbic cortex via Trk-B receptor activation to reduce drug-seeking in male rats. J. Neurosci..

[43] Pu L., Wang L., Zhang R., Zhao T., Jiang Y., Han L. (2023). Projected global trends in ischemic stroke incidence, deaths and disability-adjusted life years from 2020 to 2030. Stroke.

[44] Zhi J.F., Liao Q.H., He Y.B., Xu W.W., Zhu D.W., Shao L.H. (2022). Superior treatment efficacy of neuromodulation rehabilitation for upper limb recovery after stroke: A meta-analysis. Expert. Rev. Neurother..

[45] Porter B.A., Khodaparast N., Fayyaz T., Cheung R.J., Ahmed S.S., Vrana W.A., Rennakar R.L., Kilgard M.P. (2012). Repeatedly pairing vagus nerve stimulation with a movement reorganizes primary motor cortex. Cereb. Cortex.

[46] Borland M.S., Vrana W.A., Moreno N.A., Fogarty E.A., Buell E.P., Vanneste S., Kilgard M.P., Engineer C.T. (2019). Pairing vagus nerve stimulation with tones drives plasticity across the auditory pathway. J. Neurophysiol..

[47] Jelinek M., Lipkova J., Duris K. (2014). Vagus nerve stimulation as immunomodulatory therapy for stroke: A comprehensive review. Exp. Neurol..

[48] Bremner J.D., Wittbrodt M.T., Gurel N.Z., Shandhi M.H., Gazi A.H., Jio Y., Levantsevych O.M., Huang M., Beckwith J., Herring I. (2021). Transcutaneous cervical vagal nerve stimulation in patients with posttraumatic stress disorder (PTSD): A pilot study of effects on PTSD symptoms and interleukin-6 response to stress. J. Affect. Disord. Rep..

[49] Rush A.J., Marangell L.B., Sackeim H.A., George M.S., Brannan S.K., Davis S.M., Howland R., Kling M.A., Rittberg B.R., Burke W.J. (2005). Vagus nerve stimulation for treatment resistant depression: A randomized, controlled acute phase trial. Biol. Psychiatry.

[50] Dawson J., Liu C.Y., Francisco G.E., Cramer S.C., Wolf S.L., Dixit A., Alexander J., Ali R., Brown B.L., Feng W. (2021). Vagus nerve stimulation paired with rehabilitation for upper limb motor function after ischemic stroke (VNS-REHAB): A randomized, blinded, pivotal, device trial. Lancet.

[51] Revesz D., Rydenhag B., Ben-Menachem E. (2016). Complications and safety of vagus nerve stimulation: 25 years of experience at a single center. J. Neurosurg. Pediatr..

[52] Capone F., Miccinilli S., Pellegrino G., Zollo L., Simonetti D., Bressi F., Florio L., Ranieri F., Falato E., Di Santo A. (2017). Transcutaneous vagus nerve stimulation combined with robotic rehabilitation improves upper limb function after stroke. Neural Plast..

[53] Redgrave J.N., Moore L., Oyekunle T., Ebrahim M., Falidas K., Snowdon N., Ali A., Majid A. (2018). Transcutaneous auricular vagus nerve stimulation with concurrent upper limb repetitive task practice for poststroke motor recovery: A pilot study. J. Stroke Cerebrovasc. Dis..

[54] Wu D., Ma J., Zhang L., Wang S., Tan B., Jia G. (2020). Effect and safety of transcutaneous auricular vagus nerve stimulation on recovery of upper limb motor function in subacute ischemic stroke patients: A randomized pilot study. Neural Plast..

[55] Baig S.S., Falidas K., Laud P.J., Snowdon N., Farooq M.U., Ali A., Majid A., Redgrave J.N. (2019). Transcutaneous Auricular Vagus Nerve Stimulation with Upper Limb Repetitive Task Practice May Improve Sensory Recovery in Chronic Stroke. J. Stroke Cerebrovasc. Dis..

[56] Peng X., Baker-Vogel B., Sarhan M., Short E.B., Zhu W., Liu H., Kautz S., Badran B.W. (2023). Left or right ear? A neuroimaging study using combined taVNS/fMRI to understand the interaction between ear stimulation target and lesion location in chronic stroke. Brain Stimul..

[57] Tsao C.W., Aday A.W., Almarzooq Z.I., Alonso A., Beaton A.Z., Bittencourt M.S., Boehme A.K., Buxton A.E., Carson A.P., Commodore-Mensah Y. (2022). Heart Disease and Stroke Statistics-2022 Update: A report from the American Heart Association. Circulation.

[58] Wade D.T., Langton-Hewer R., Wood V.A., Skilbeck C.E., Ismail H.M. (1983). The hemiplegic arm after stroke: Measurement and recovery. J. Neurol. Neurosurg. Psychiatry.

[59] Carod-Artal F.J., Egido J.A. (2009). Quality of life after stroke: The importance of a good recovery. Cerebrovasc. Dis..

[60] Mayo N.E., Wood-Dauphinee S., Côté R., Durcan L., Carlton J. (2002). Activity, participation, and quality of life 6 months poststroke. Arch. Phys. Med. Rehabil..

[61] Mccabe J., Monkiewicz M., Holcomb J., Pundik S., Daly J.J. (2015). Comparison of robotics, functional electrical stimulation, and motor learning methods for treatment of persistent upper extremity dysfunction after stroke: A randomized controlled trial. Arch. Phys. Med. Rehabil..

[62] Ward N.S., Brander F., Kelly K. (2019). Intensive upper limb neurorehabilitation in chronic stroke: Outcomes from the Queen Square programme. J. Neurol. Neurosurg. Psychiatry.

[63] Wolf S.L., Winstein C.J., Miller J.P., Taub E., Uswatte G., Morris D., Giuliani C., Light K.E., Nichols-Larsen D., Excite Investigators (2006). Effect of constraint-induced movement therapy on upper extremity function 3 to 9 months after stroke: The EXCITE randomized clinical trial. JAMA.

[64] Hays S.A., Rennaker R.L., Kilgard M.P. (2013). Targeting plasticity with vagus nerve stimulation to treat neurological disease. Prog. Brain Res..

[65] Engineer N.D., Kimberley T.J., Prudente C.N., Dawson J., Tarver W.B., Hays S.A. (2019). Targeted vagus nerve stimulation for rehabilitation after stroke. Front. Neurosci..

[66] Lemon R.N. (2008). Descending pathways in motor control. Ann. Rev. Neurosci..

[67] Meyers E.C., Solorzano B.R., James J., Ganzer P.D., Lai E.S., Rennaker R.L., Kilgard M.P., Hays S.A. (2018). Vagus nerve stimulation enhances stable plasticity and generalization of stroke recovery. Stroke.

[68] Loerwald K.W., Borland M.S., Rennaker R.L.I.I., Hays S.A., Kilgard M.P. (2018). The interaction of pulse width and current intensity on the extent of cortical plasticity evoked by vagus nerve stimulation. Brain Stimul..

[69] Ben-Menachem E., Mañon-Espaillat R., Ristanovic R., Wilder B., Stefan H., Mirza W., Tarver W.B., Wernicke J.F. (1994). Vagus nerve stimulation for treatment of partial seizures: 1. A controlled study of effect on seizures. Epilepsia.

[70] Hays S.A., Khodaparast N., Hulsey D.R., Ruiz A., Sloan A.M., Rennaker R.L., Kilgard M.P. (2014). Vagus nerve stimulation during rehabilitative training improves functional recovery after intracerebral hemorrhage. Stroke.

[71] Burger A.M., D’Agostini M., Verkuil B., Van Diest I. (2020). Moving beyond belief: A narrative review of potential biomarkers for transcutaneous vagus nerve stimulation. Psychophysiology.

[72] Bucksot J.E., Morales Castelan K., Skipton S.K., Hays S.A. (2020). Parametric characterization of the rat Hering-Breuer reflex evoked with implanted and non-invasive vagus nerve stimulation. Exp. Neurol..

[73] Peuker E.T., Filler T.J. (2002). The nerve supply of the human auricle. Clin. Anat..

[74] Planitzer U., Hammer N., Bechmann I., Glätzner J., Löffler S., Möbius R., Tillmann B.N., Weise D., Winkler D. (2017). Positional relations of the cervical vagus nerve revisited. Neuromodulation.

[75] Korupolu R., Miller A., Park A., Yozbatiran N. (2024). Neurorehabilitation with vagus nerve stimulation: A systematic review. Front. Neurol..

[76] Kimberley T.J., Prudente C.N., Engineer N.D., Dickie D.A., Bisson T.A., Van De Winckel A. (2023). Vagus nerve stimulation paired with mobility training in chronic ischemic stroke: A case report. Phys. Ther..

[77] Morrison R.A., Hays S.A., Kilgard M.P. (2021). Vagus nerve stimulation as a potential adjuvant to rehabilitation for post-stroke motor speech disorders. Front. Neurosci..

[78] Michaelsen S.M., Dannenbaum R., Levin M.F. (2006). Task-specific training with trunk restraint on arm recovery in stroke: Randomized control trial. Stroke.

[79] Khodaparast N., Kilgard M.P., Casavant R., Ruiz A., Qureshi I., Ganzer P.D., Rennaker R.L., Hays S.A. (2016). Vagus nerve stimulation during rehabilitative training improves forelimb recovery after chronic ischemic stroke in rats. Neurorehabil. Neural Repair.

